# Mechanism of Xinfeng Capsule on Adjuvant-Induced Arthritis via Analysis of Urinary Metabolomic Profiles

**DOI:** 10.1155/2016/5690935

**Published:** 2016-02-18

**Authors:** Hui Jiang, Jian Liu, Ting Wang, Jia-rong Gao, Yue Sun, Chuan-bing Huang, Mei Meng, Xiu-juan Qin

**Affiliations:** ^1^Department of Pharmacy, The First Affiliated Hospital of Anhui University of Chinese Medicine, 117 Meishan Road, Hefei 230031, China; ^2^College of Basic Medicine, Anhui Medical University, 81 Meishan Road, Hefei 230022, China; ^3^College of Pharmacy, Anhui University of Chinese Medicine, 103 Meishan Road, Hefei 230038, China

## Abstract

We aimed to explore the potential effects of Xinfeng capsule (XFC) on urine metabolic profiling in adjuvant-induced arthritis (AA) rats by using gas chromatography time-of-flight mass spectrometry (GC-TOF/MS). GC-TOF/MS technology was combined with multivariate statistical approaches, such as principal component analysis (PCA), partial least squares discriminant analysis (PLS-DA), and orthogonal projections to latent structures discriminant analysis (OPLS-DA). These methods were used to distinguish the healthy group, untreated group, and XFC treated group and elucidate potential biomarkers. Nine potential biomarkers such as hippuric acid, adenine, and L-dopa were identified as potential biomarkers, indicating that purine metabolism, fat metabolism, amino acid metabolism, and energy metabolism were disturbed in AA rats. This study demonstrated that XFC is efficacious for RA and explained its potential metabolomics mechanism.

## 1. Introduction

Rheumatoid arthritis (RA) is a common chronic systematic autoimmune disease that is characterized by inflammation of synovial tissue. It is often recurrent and protracted, painful, and results in stiff and damaged bone and cartilage. RA has a high morbidity and disability rate and seriously affects patient's quality of life [1, 2].

Under the guidance of the Xin'an medical theory, Xinfeng capsule (XFC, Anhui medicine system Z20050062) is a Traditional Chinese Medicine (TCM) prescription with a long history used in the clinic for the treatment of RA [3–5], which has been registered in China with patent certificates (ZL 201310011369.8 and ZL201410101266.5). XFC is composed of* Coix seed* (Yiyiren),* Scolopendra* (Wugong),* Astragalus* (Huangqi), and* Tripterygium wilfordii* (Leigongteng). Methods of extraction and preparation of the XFC formulation were studied in our previous experiments; they showed that the extraction method was appropriate, simple, and feasible; quality control data were available. A proper chemical profile HPTLC for the evaluation of* Coix seed*,* Scolopendra*,* Astragalus*, and* Tripterygium wilfordii* were already studied [6]. In our previous study, XFC had a good effect on relieving the degree of joint pain, alleviating morning stiffness and enhancing grip strength in RA, without obvious adverse reactions [7, 8]. However, the metabolomics mechanism of Xinfeng Capsule that cure RA is unclear.

Metabolomics is a technology in which all endogenous metabolites are analyzed via high-throughput detection and data processing to obtain potential biomarkers [9]. This technology can elucidate the status of an organism at a specific time and under a specific environment [10]. Metabolomics has comprehensive analysis and can dynamically operate, which is conducive to the study of TCM that have complex mixtures of metabolites and compounds [11]. Therefore, metabolomics can be used to explore the relationship between changes in the physiological state of a biological system and potential biomarkers [12].

In this study, we used a metabolomic approach based on gas chromatography time-of-flight mass spectrometry (GC-TOF/MS) technology to find potential endogenous biomarkers in urine. We aimed to explore the therapeutic effects and metabolic mechanism of XFC in a model of RA.

## 2. Materials and Methods

### 2.1. Experimental Animal

The protocol was approved by the Committee on the Ethics of Animal Experiments of Anhui University of Chinese medicine (Permit Number: 2012AH-038-01). All surgery was performed under sodium pentobarbital anesthesia, and all efforts were made to minimize suffering. Adult male Sprague-Dawley (SD) rats (180–200 g) of specified pathogen free (SPF) were purchased from Anhui Medical University Laboratory Animal Center. All animals were placed in standard cages under controlled temperature (around 22°C) and relative humidity (40%–60%) conditions, with the same amount of food and water at a regular daily time.

### 2.2. Drugs and Reagents

Internal standard: L-2-Chlorophenylalanine (CAS: 103616-89-3) was purchased from Shanghai Heng Bo Biological Technology Co., China. N,O-bis-trimethylsilyl-trifluoroacetamide (BSTFA) with 1% trimethylchlorosilane (TMCS) was purchased from REGIS Technologies Inc., USA. Urease from Canavalia ensiformis (Jack bean), Type III, powder, was purchased from Sigma company. Paraformaldehyde solution was purchased from Sinopharm Chemical Reagent Co. Ltd. XFC was provided by the First Affiliated Hospital of Anhui University of Chinese Medicine (Batch number: 2012100407, Hefei, China).

### 2.3. Instruments and Equipment

Instruments and equipment are as follows: GC chromatograph: Agilent 7890A, Agilent, USA; Mass spectrometer: LECO Chroma TOF PEGASUS 4D, LECO, USA; GL-20A Automatic high speed refrigerated centrifuge (Hunan Instruments Plant centrifuge plant); −80°C Ultra-Low Temperature Freezer-Uplight type (Sanyo company); 37°C incubator (Hubei Huangshi Medical Instruments Factory).

### 2.4. Induction, Treatment, and Measurement of Adjuvant-Induced Arthritis (AA)

SD rats were adaptively fed for one week. All rats were divided randomly into three groups: healthy group, untreated group, and XFC treated group, with 8 rats in each group. The untreated group and XFC treated group were established with a single intracutaneous injection of 0.1 mL of complete Freund's adjuvant (CFA) into the right hind metatarsal footpad [13]. The healthy group was given liquid paraffin for comparison at the same time. From day 19 after injection of CFA, XFC (3 g/kg) were given by gavage once a day for 30 days in the XFC treated group. Rats in the healthy and untreated groups were given 0.9% saline. Urine was collected 12 h after the last administration and kept at −80°C. Two different clinical parameters, paw swelling and arthritis index (AI), were used to assess the therapeutic effects of XFC on AA rats. The volume of the left hind paw was measured with a PV-200 volume meter (Chengdu Taimeng Technology Co., Chengdu, China) before immunization (normal value, day 0) and after the last administration (day 30). Secondary paw swelling (*E*) was calculated with the formula *E*(%) = (*V*_*t*_ − *V*_0_)/*V*_0_ × 100%, where *V*_0_ and *V*_*t*_ represent the volume of the paw before modeling and after modeling, respectively [14]. For evaluation of AA, the arthritis severity was recorded on a four-point scale, where 0 indicates no swelling; 1 point indicates mind swelling; 2 points indicate slight swelling of wrist or ankle joints; 3 points indicate severe swelling of the entire joint and paw; and 4 points indicate ankylosis or deformed paws [15].

### 2.5. Histological Examination

All rats were sacrificed at 12 hours after the last administration. The knee was removed and placed in 4% paraformaldehyde solution. Histological changes were observed after pathological HE staining using conventional optical microscopes. Two blinded observers evaluated cartilage and bone destruction by cellular infiltration, synovial proliferation, pannus formation, and cartilage erosion, using the following scoring system: cellular infiltration: Grade 0, no changes; Grade 1, few focal infiltrates; Grade 2, extensive focal infiltrates; and Grade 3, extensive infiltrates invading the capsule with aggregate formation; synovial proliferation: Grade 0, proliferation was absent; Grade 1, proliferation was mild with two to four layers of reactive synoviocytes; Grade 2, proliferation was moderate with four plus layers of reactive synoviocytes, increased mitotic activity, and mild or absent synovial cell invasion of adjacent bone and connective tissue; and Grade 3, proliferation was severe and characterized by invasion and effacement of joint space and adjacent cartilage, bone, and connective tissue; pannus formation: Grade 0, no changes; Grade 1, pannus formation at up to two sites; Grade 2, pannus formation at up to four sites, with infiltration or flat overgrowth of joint surface; and Grade 3, pannus formation at more than four sites or extensive pannus formation at two sites; cartilage erosion: Grade 0, no changes; Grade 1, superficial, localized cartilage degradation in more than one region; Grade 2, localized deep cartilage degradation; and Grade 3, extensive deep cartilage degradation at several locations [16].

### 2.6. Sample Preparation

Urine samples were thawed at room temperature and vortexed for 15 s, after which 100 *μ*L of the urine was transferred into 1.5 mL Eppendorf tubes and subsequently 10 *μ*L of urease suspension (160 mg/mL in water) was added and vortexed for 10 s. Up to 900 *μ*L of a mixture of methanol and chloroform (3/1, vol/vol) and 50 *μ*L of L-2-Chlorophenylalanine (0.2 mg/mL) as internal standards were added to each sample and vortexed for 10 min and subsequently centrifuged at 12,000 rpm for 10 min at 4°C. Transfer the supernatant (approximately 0.35 mL) into new 2 mL GC-TOF/MS glass vials. The collected supernatant fluid was concentrated to complete dryness in a vacuum concentrator without heating. 80 *μ*L methoxymethyl amine salt was added, which aimed to dry up metabolites at 37°C for 2 h in an oven after mixing and sealing. The dried samples were then derivatized with the addition of 100 *μ*L bis-(trimethylsilyl)-trifluoroacetamide with 1% trimethylchlorosilane into each vial. The mixture was left to react for 1 h in an oven at 70°C. After incubation, the samples were again vortexed for 1 min in preparation for GC-TOF/MS analysis.

### 2.7. GC-TOF/MS Analysis

GC-TOF/MS analysis was performed using an Agilent 7890 gas chromatograph system coupled with a Pegasus HT time-of-flight mass spectrometer. The system utilized a DB-5MScapillary column coated with 5% diphenyl cross-linked with 95% dimethyl polysiloxane (30 m × 250 *μ*m inner diameter, 0.25 *μ*m film thickness; J&W Scientific, Folsom, CA, USA). A 1 *μ*L aliquot of the analyte was injected in splitless mode. Helium was used as the carrier gas, the front inlet purge flow was 3 mL/min, and the gas flow rate through the column was 20 mL/min. The initial temperature was kept at 50°C for 1 min, then raised to 330°C at a rate of 10°C/min, and then kept for 5 min at 330°C. The injection, transfer line, and ion source temperatures were 280, 280, and 220°C, respectively. The energy was −70 eV in electron impact mode. The mass spectrometry data were acquired in full-scan mode with the *m*/*z* range of 85–600 at a rate of 20 spectra per second after a solvent delay of 366 s.

### 2.8. Data Analysis

Chroma TOF4.3X software of LECO Corporation and LECO-Fiehn Rtx5 database were used for raw peaks exacting, the data baselines filtering and calibration of the baseline, peak alignment, deconvolution analysis, peak identification, and integration of the peak area [17]. The resulting normalized peak intensities were analyzed by PCA, PLS-DA, and OPLS using the DEMO (Umetrics AB, Umea, Sweden). Data was processed by Spss 17.0 version (Spss, Inc., Chicago, IL), and statistical analyses were handled using Student's *t*-test.

## 3. Results

### 3.1. Effects of XFC on Secondary Paw Swelling and Arthritis Index (AI)

Before intragastric administration of XFC, compared with the healthy group, rats in the untreated group had significantly more secondary paw swelling and higher arthritis index (*p* < 0.01). The secondary paw swelling and arthritis index in the XFC treated group were similar to those of untreated group. After intragastric administration of XFC for 30 days, the AA rats had little secondary paw swelling and a lower arthritis index in the XFC treated group, compared with the untreated group (*p* < 0.01). This result indicated that XFC could inhibit secondary inflammation in AA rats (Table 1).

### 3.2. Impact of XFC on Histopathology

**Figure 1:**
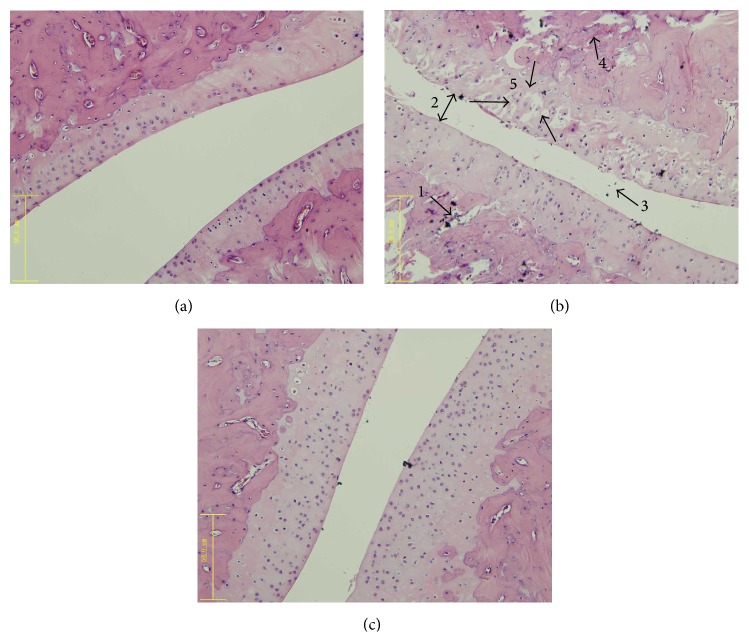
Effects of XFC on histopathologic changes of the AA rats. Photomicrographs of representative histopathologic specimens of left hind joints in SD rats taken from healthy group (a), untreated group (b), and XFC treated group (c). 1: inflammatory cell infiltration; 2: joint-space narrowing; 3: neutrophils infiltration; 4: pannus formation; 5: cartilage erosion. Original magnification 400x.

**Figure 2:**
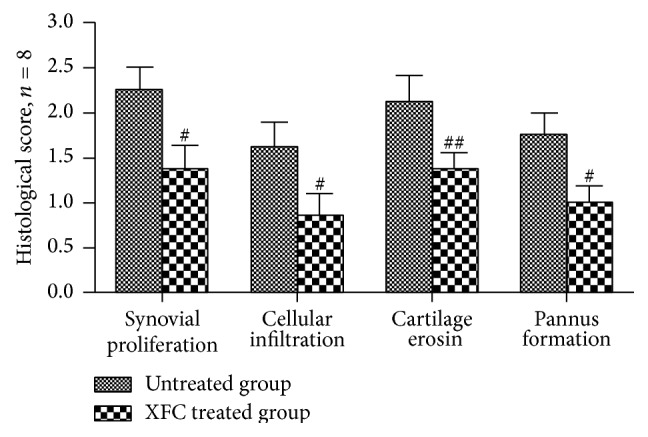
Histological score of the AA rats, ^#^ *p* < 0.05, ^##^ *p* < 0.01 compared with untreated group.

Under HE staining, the healthy group had normally arranged synovial cells, no synovial hyperplasia, no inflammatory cell infiltration, and no cartilage erosion. The untreated group had local thickening of the surrounding synovium tissue, inflammatory cell infiltration accompanied by spalling pannus formation, joint-space narrowing, and some cartilage erosion. The XFC treated group had less pathological tissue injury than that of the untreated group to varying degrees (Figures 1 and 2).

### 3.3. Total Ion Chromatogram of Urine Samples

**Figure 3:**
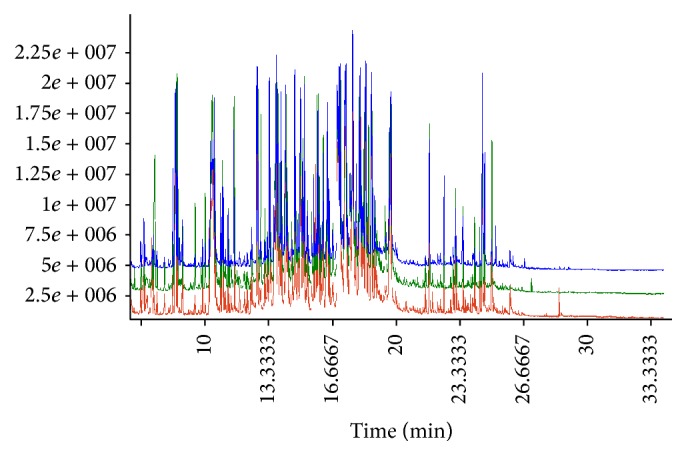
TIC chromatogram of urine samples in AA rats; the brown line represents healthy group; the green line represents untreated group; the blue line represents XFC treated group.

The GC-TOF/MS TIC chromatograms of urine samples from the healthy group, untreated group, and XFC treated group are shown in Figure 3. Based on LECO-Fiehn Rtx5 database, approximately 1166 metabolites were identified. After using Chroma TOF4.3X software to correct the blank value, eliminate noise, and correct an internal standard, 914 metabolites were identified. The horizontal axis represents the time at which metabolites occur; each peak represents the relative the metabolite. In order to illustrate the differences of the metabolic profiles, GC-TOF/MS spectra were further pretreated and a pattern recognition analysis was carried out.

### 3.4. Principal Component Analysis (PCA) of Urine Samples

**Figure 4:**
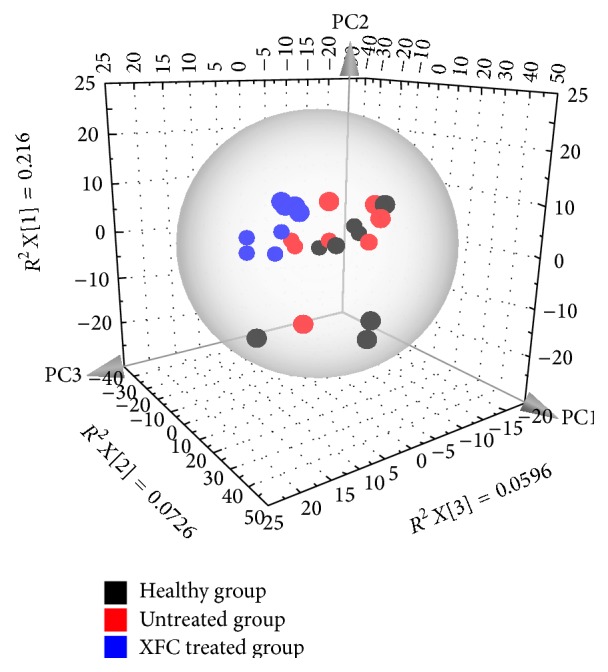
PCA score plots of adjuvant-induced arthritis in rats urine metabolic profiling of healthy group, untreated group, and XFC treated group using GC-TOF/MS.

PCA, the most-commonly used algorithm in metabolomic studies, was employed to process the GC-TOF/MS data using the software developed in SIMCA-P (Umetrics, Umea, Sweden). The simultaneous comparison of a large number of complex variables was facilitated by a dimensional reduction via three-dimensional mapping procedures and the output displayed with score plots, which represented the distribution of samples in multivariate space [18]. In PCA score plots (Figure 4), the urine samples from each group did not completely separate, which will need further analysis.

### 3.5. Partial Least Squares Discriminant Analysis (PLS-DA) of Urine Samples

**Figure 5:**
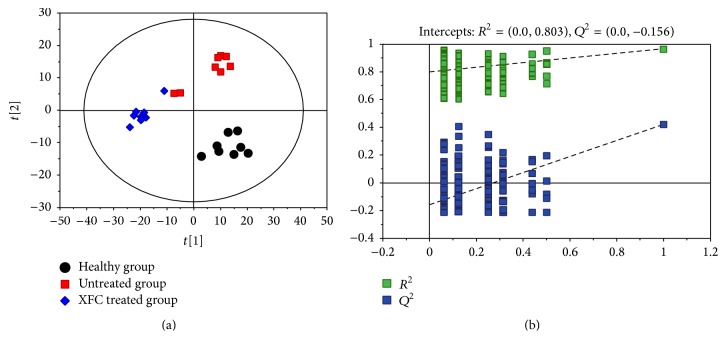
PLS-DA based on the adjuvant-induced arthritis date of urine. (a) shows the separation for the PLS-DA scores of healthy, untreated, and XFC treated groups. (b) represents validation of PLS-DA model (using 200 random permutations).

Because PCA failed to completely separate, PLS-DA analysis was performed to achieve greater separation between the groups and enhance the identification of metabolites. PLS-DA used Ctr (mean-centered scaling) to process the data scale conversion mode, where the *x*- and *y*-axes represent the first and second principle components. The leave-one-out cross-validation method was used to assess the actual performance of the established model [19]. *R* ^2^ is the fraction of the Sum of Squares (SS) explained by the model, which represents model fitness; *Q* ^2^ is the fraction of *Y* variation predicted by the *X* model in that component, which represents predictive ability. These were obtained after using cross validation to evaluate validity of the model. After PLS-DA analysis, the healthy, untreated, and XFC treated groups completely separated. *R* ^2^ *Y* = 0.997 and *Q* ^2^ *Y* = 0.553, which indicate that the model has a perfect fitting and has reliable predictive ability. After the randomization (*n* = 200), we obtained *R* ^2^ = 0.803 and *Q* ^2^ = −0.156, indicating that both of these PLS-RA models demonstrated good performances [20]. The results are shown in Figure 5.

### 3.6. Orthogonal Partial Least-Squares Discriminant Analysis (OPLS-DA) of Urine Samples

**Figure 6:**
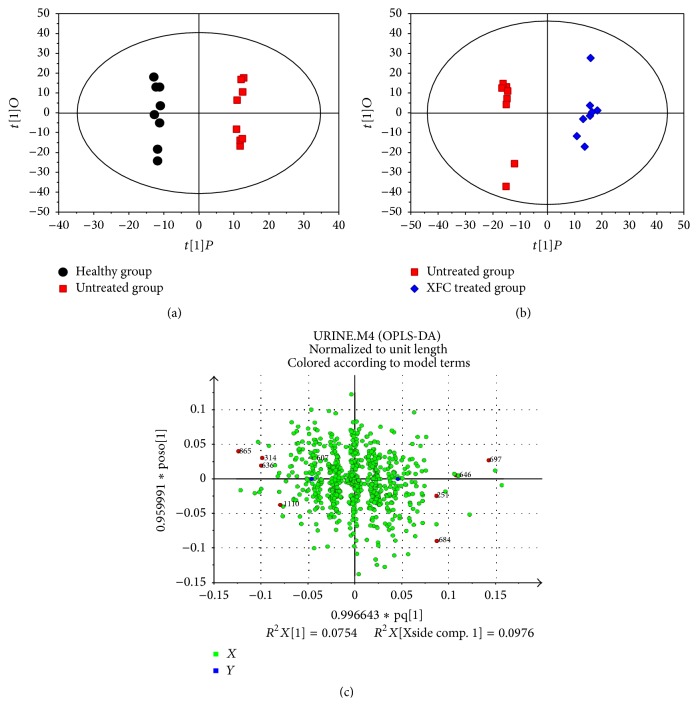
OPLS-DA of urine metabolic profiling of healthy group versus untreated group (a); OPLS-DA of urine metabolic profiling of untreated group versus XFC treated group (b); OPLS-DA variable loading plot (c).

OPLS-DA is a model for orthogonal correction in which the orthogonal signal correction can be filtered out with uncorrelated orthogonal variable information. This method retains only correlated orthogonal variable information and improves the accuracy of discrimination. OPLS-DA was performed using SIMCA-P software, which maximizes the internal differences of the model between different groups [21]. OPLS-DA used Ctr (mean-centered scaling) to process the data scale conversion mode where the *X*- and *Y*-axes represent first and second principle components, respectively. After OPLS-DA analysis, the urine metabolomes in each group were clustered. This result suggests that the metabolic pattern in AA rats was significantly altered and that XFC intervention can affect the metabolism of AA rats. The results are shown in Figures 6(a) and 6(b).

We obtained a load diagram from OPLS-DA. Each dot represents a metabolite. Dots near the center represent small difference in model classification, and dots far away from the center were considered to contribute more to the model classification. Red dots represent the nine potential biomarkers in our test. Results are shown in Figure 6(c).

### 3.7. Potential Biomarkers from Urine Samples

OPLS-DA filtered out unrelated orthogonal signals, so more reliable potential biomarkers were obtained. VIP is a parameter which summarizes the importance of the *X*-variables, both for the *X* and *Y* models, is called the variable influence on projection, which estimates the importance of each variable in the projection used in a OPLS-DA model. A variable with a VIP score close to or greater than 1 can be considered important in a given model. The VIP value and Student's *t*-test of the *p* value were used to reflect the importance of the metabolites. Variables with VIP > 1 and *p* < 0.05 were selected as potential biomarkers for further statistical analysis using SPSS version 17.0 (Spss Inc., Chicago, IL, USA). Nine potential biomarkers were observed as compared with that of healthy group (*p* < 0.05), which were correlated with adjuvant-induced arthritis. After the treatment of XFC, the levels of the 2,2-dimethylsuccinic acid, phenaceturic acid, L-dopa, and 1,4-dihydroxy-2-naphthoic acid were changed significantly (*p* < 0.05). The levels of dehydroshikimic acid, tartronic acid, adenine, hippuric acid, and melibiose were trends that have tendency toward healthy group, but there was no significant difference. Results are shown in Table 2.

**Figure 7:**
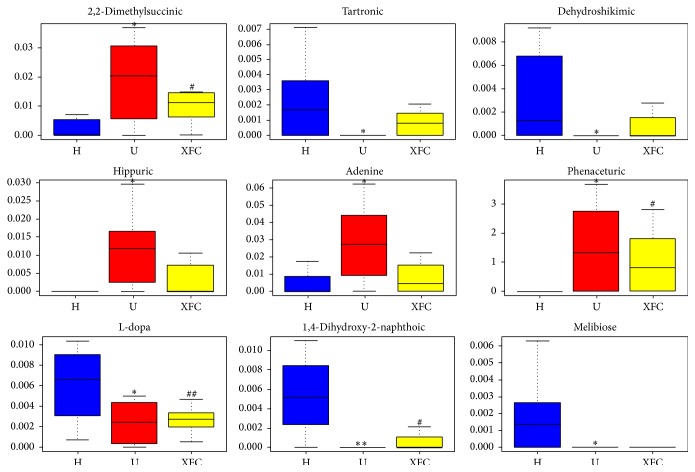
Box plots of data in urine samples from healthy group (H), untreated group (U), and XFC treated group (XFC). ^*∗*^ *p* < 0.05 and ^*∗∗*^ *p* < 0.01 compared with healthy group; ^#^ *p* < 0.05 compared with untreated group; ^##^ *p* < 0.01 compared with untreated group.

To better visualize the changes in the potential biomarkers, box plots were created using straightforward statistical graphics to describe the distribution of several sets of quantitative data and compare their differences. The height of the box represents the interquartile range, the horizontal line represents the median, and the extensions up and down at the ends of the thread represent the maximum and the minimum, shown in Figure 7.

### 3.8. Correlation Network Analysis of Potential Biomarkers

**Figure 8:**
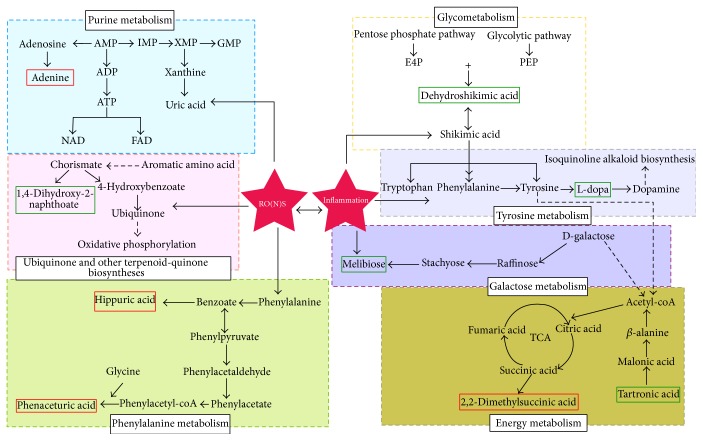
Correlation networks of all the potential biomarkers in response to RA. Nine potential biomarkers were used to explain the possible metabolic pathways. Metabolites with red box were significantly increased in untreated group compared to healthy group. Metabolites with blue box were significantly decreased in untreated group compared to healthy group.

To illustrate the links in the potential biomarker metabolic pathways, we used pathophysiology, biochemistry, physiology, and the Human Metabolome Database to provide quantitative and metabolic information on organism metabolites. We also referred to the relevant literature. The nine potential biomarkers are involved in purine metabolism, fat metabolism, amino acid metabolism, and energy metabolism. Therefore, we constructed a network of metabolic pathways of potential biomarkers, shown in Figure 8.

## 4. Discussion

AA, a T-cell-mediated chronic inflammatory stress, is a well-established in vivo model that has been used in numerous studies to investigate the pathogenesis of RA and for identification of potential therapeutic targets [22]. After intragastric administration of XFC for 30 days, there was significantly less joint swelling in AA rats (*p* < 0.01) and less histological damage, compared with model rats. Therefore, XFC has a moderate therapeutic effect on AA rats.

Metabolomics focuses on changes in biological fluids to indicate changes in a biological system [23]. In recent years, metabolomics has been used to analyze biological differences in metabolic profiles to identify potential biomarkers. These differences can elucidate the overall function of organisms at specific times and in specific environments. GC-TOF/MS, liquid chromatography-mass spectrometry, and nuclear magnetic resonance are commonly used in metabolomics [24–26]. GC-TOF/MS is a variety of features including simplicity, reproducibility, high separation efficiency, high sensitivity, standard spectral library, and quantifiable metabolites in metabolomics [27]. We used GC-TOF/MS and found nine potential biomarkers. The biomarkers are mainly involved in the glucose, fat, amino acid, and energy metabolism pathways. After intragastric administration of XFC, the levels of the 2,2-dimethylsuccinic acid, phenaceturic acid, L-dopa, and 1,4-dihydroxy-2-naphthoic acid were changed significantly in the XFC treated group (*p* < 0.05). The levels of dehydroshikimic acid, hippuric acid, and melibiose are trends that have tendency toward healthy group, but there was no significant difference.

Studies have shown that free radical oxidative damage plays an important role in the pathogenesis of RA [28]. Endogenous or exogenous stimuli can result in metabolic abnormalities, which can produce excessive reactive oxygen species. When the amino acid supply is inadequate, a reduction in antioxidant enzymes results in disordered balance between prooxidant/antioxidants [29]. This imbalance eventually leaves the body in a state of oxidative stress. Reactive oxygen species can damage DNA, lipids, proteins, and other biological molecules, which can result in energy metabolism disorders. However, energy consumption will also produce acidic substances and excessive active oxygen radicals, which further aggravate tissue damage and cause inflammation. Therefore, oxidative stress and inflammation play a key role in RA [30].

Metabolomic analysis of changes in the urine of AA rats improves our understanding of significant metabolic variations during RA. Hippuric acid and phenaceturic acid are secondary metabolites of fatty acids. During RA, excessive oxygen free radicals are produced in the mitochondria, which accelerate fat mobilization and eventually significantly increase the levels of fatty acid secondary metabolites. We found higher levels of hippuric acid and phenaceturic acid in the urine of AA rats, which implies that RA is associated with lipid metabolism dysfunction [31, 32]. The XFC treated group had significantly lower level of phenaceturic acid than that of the untreated group. Hippuric acid content has no significant difference in the XFC treated group but has a tendency towards healthy group. This result implies that XFC can regulate fat metabolism and reduce oxygen free radical generation in the mitochondria.

L-dopa is a tyrosine oxidation product that is generated from tyrosine hydroxylase. Tyrosine is the major metabolite of phenylalanine, which are both amino acids. During RA, excessive oxidative stress and inflammation inhibit amino acid intake or slow their synthesis, which affects the generation of articular cartilage protein [31–33]. The untreated group had lower levels of L-dopa compared with the healthy group, which indicates that amino acid metabolism was disrupted. The XFC treated group had higher levels of L-dopa compared with the untreated group, which implies that XFC can regulate amino acid metabolism and improve joint damage.

Lower levels of tartronic acid and higher levels of 2,2-dimethylsuccinic acid were observed in the untreated group, which indicates dysfunction of the tricarboxylic acid cycle (TCA) probably because of disturbed metabolism in the chondrocytes and cartilage. 2,2-Dimethylsuccinic acid is a succinic methylated product and succinic acid is an important metabolic intermediate in the TCA. The TCA is the final metabolic pathway for the three major nutrients, which are carbohydrates, lipids, and amino acids. The XFC treated group had significantly reduced levels of 2,2-dimethylsuccinic acid compared with the untreated group. These changes suggest that XFC has a regulatory role on the TCA.

1,4-Dihydroxy-2-naphthoic acid is an aromatic amino acid that is the product of various enzymes and is involved in ubiquinone and other terpenoid and quinone biosyntheses. 1,4-Dihydroxy-2-naphthoic acid was lower in the untreated group, probably because there were too many oxygen free radicals, which resulted in increased ubiquinone production and strengthened oxidative phosphorylation. These changes resulted in more ATP [34]. The XFC treated group had higher 1,4-dihydroxy-2-naphthoic acid levels compared with the untreated group, indicating improved energy metabolism.

Melibiose is composed of a galactose and glucose. Not only can a stachyose be decomposed into a melibiose but also it generates manninotriose enhanced immune substances [35], which improve immunity to fight inflammation consistent with prophase study [36]. During RA, abnormalities in galactose metabolism lead to lower levels of melibiose and manninotriose. The XFC group had higher melibiose levels, which suggest that XFC can adjust disordered galactose metabolism and the immune system to reduce inflammation. We found lower concentrations of dehydroshikimic acid in the urine of AA rats as compared with normal controls. Abnormalities in urinary dehydroshikimic acid are generally believed to be associated with disorder of glycolytic pathway and pentose phosphate pathway. Shikimic acid has anti-inflammatory, analgesic, and antiviral effects in accord with reference [37]. The levels of melibiose and dehydroshikimic acid have no significant difference in the XFC treated group but have a tendency towards healthy group, which suggested that XFC maybe achieve a certain effect to regulate the disorder of glycometabolism and reinforced the anti-inflammatory capacity.

Tartronic acid is converted to *β*-alanine that is a component of coenzyme A, which plays an important role in the TCA. Adenine can generate uric acid during RA; the elevated levels of adenine lead to high levels of uric acid, which aggravates cartilage damage. In addition, adenine participating cofactors include nicotinamide adenine dinucleotide (NAD) and flavin adenine dinucleotide (FAD). These compounds are progressively oxidized by the respiratory chain to generate ATP. We hypothesize that oxidative stress leads to reduction of functional capacity of mitochondria, which leads to an energy metabolism disorder [34]. Our results show that the levels of tartronic acid and adenine had no significant difference in XFC treated group, implying that XFC did not regulate them for treatment.

## 5. Conclusions

We used a GC-TOF/MS metabolomics method to comprehensively and efficiently elucidate changes in the metabolites AA rats. Using a variety of multivariate statistical methods such as PCA, PLS-DA, and OPLS-DA, nine potential biomarkers were identified. These biomarkers are mainly associated with purine metabolism, fat metabolism, amino acid metabolism, and energy metabolism.

After administration of XFC, the levels of the 2,2-dimethylsuccinic acid, phenaceturic acid, L-dopa, and 1,4-dihydroxy-2-naphthoic acid were changed significantly (*p* < 0.05). The levels of dehydroshikimic acid, hippuric acid, and melibiose had no significant difference, but there are trends that have tendency toward healthy group. These findings show that GC-TOF/MS-based urine metabolic profiling can provide reliable results and be used in modern TCM research. This technique has potential application in identification effective compounds to treat RA.

## Figures and Tables

**Table 1:** Effects of XFC on paw swelling and arthritis index in AA rats.

Group	Paw swelling (%)		Arthritis index	
	Day 0	Day 30	Day 0	Day 30
Healthy	34.79 ± 10.56	42.21 ± 10.32	0.00 ± 0.00	0.00 ± 0.00
Untreated	61.19 ± 12.92^*∗∗*^	78.15 ± 12.28^*∗∗*^	7.50 ± 1.60^*∗∗*^	8.63 ± 2.07^*∗∗*^
XFC treated	64.67 ± 11.99^*∗∗*^	56.78 ± 17.67^##^	7.75 ± 1.83^*∗∗*^	5.38 ± 1.59^*∗∗*##^

Note: day 0 shows paw swelling and arthritis index before intragastric administration of XFC; day 30 shows paw swelling and arthritis index on day 30 after intragastric administration of XFC. Data represent the mean ± SD (*n* = 8). ^*∗∗*^ *p* < 0.01 compared with healthy group; ^##^ *p* < 0.01 compared with untreated group.

**Table 2:** Potential biomarkers selected and change of trend in each group.

Number	VarID	Compound	RT	VIP	*p*_H-U_	*p*_U-X_	*p*_X-H_
1	251	2,2-Dimethylsuccinic acid	11.0616	2.70266	0.011313387	0.03490986	0.249996112
2	314	Tartronic acid	11.8754	2.94527	0.024742601	0.15475148	0.010899989
3	636	Dehydroshikimic acid	16.7414	3.05951	0.032774968	0.1022563	0.077357634
4	646	Hippuric acid	16.9083	3.21815	0.01018095	0.19488332	0.049258272
5	684	Adenine	17.5117	2.73672	0.021520896	0.59814266	0.035198717
6	697	Phenaceturic acid	17.7171	4.18817	0.016864462	0.02460963	0.503834004
7	807	L-dopa	19.405	1.31514	0.026524125	0.0252176	0.771814517
8	865	1,4-Dihydroxy-2-naphthoic acid	20.474	3.69942	0.003037716	0.00524929	0.612627294
9	1110	Melibiose	25.9579	2.33593	0.040379626	0.0682718	0.501486543

Note: VarID represents urine screening 1166 peak to the serial number of compound; RT represents retention time of compound. A variable importance in the projection (VIP) was obtained from OPLS-DA model with a threshold of 1.0; *p*_H-U_ represents *p* value by healthy group versus untreated group; *p*_U-X_ represents *p* value by untreated group versus XFC treated group; *p*_X-H_ represents *p* value by XFC treated group versus healthy group.
